# The significance of synovial biopsy in the diagnostic workup of the low-grade periprosthetic joint infection of shoulder arthroplasty

**DOI:** 10.1007/s00402-021-03932-x

**Published:** 2021-05-15

**Authors:** Moritz Mederake, Ulf Krister Hofmann, Bernd Fink

**Affiliations:** 1 Department of Orthopaedic Surgery, University Hospital Tübingen, Hoppe Seyler-Str. 3, 72076 Tübingen, Germany; 2 Department of Joint Replacement, Orthopaedic Clinic Markgröningen, Kurt-Lindemann-Weg 10, 71706 Markgröningen, Germany; 3 Department of Orthopaedic Surgery, Universtity Hospital Hamburg-Eppendorf, Martinistr. 52, 20246 Hamburg, Germany

**Keywords:** Shoulder, Prosthesis-related infections, Arthroplasty, replacement, shoulder, Biopsy, Prostheses and implants

## Abstract

**Introduction:**

A common reason for painful shoulder arthroplasties and revision surgery is a low-grade periprosthetic joint infection (PJI). Diagnosing a low-grade infection is, however, a major diagnostic challenge. This applies even more to the shoulder, which differs from other large joints in terms of clinical features and microbiological spectrum. Aim of this study was to evaluate the diagnostic value of the synovial biopsy in the diagnostic workup of low-grade PJI of the shoulder.

**Materials and methods:**

A retrospective evaluation was conducted on 56 patients receiving revision surgery on their shoulder arthroplasty. A standardized preoperative workup was performed comprising CRP value, leukocyte blood count, synovial fluid microbiological analyses and leukocyte count from joint aspiration, and five synovial biopsy samples for bacteriologic and histologic analysis obtained through an arthroscopic approach. During revision surgery, five samples of periprosthetic tissue were harvested for bacteriologic and histologic analyses. The MSIS-Criteria 2014 were used to evaluate the diagnostic results.

**Results:**

In total, 15 of 56 revised prostheses turned out as PJI (27%). When applying our diagnostic workup, we obtained a sensitivity of 67% with a specificity of 95%. When performing a subgroup analysis on those patients that had received diagnostic biopsy, a sensitivity of 100% and a specificity of 83% could be achieved. With a sensitivity and specificity of 90% and 83%, respectively, the biopsy is the single method with the highest diagnostic value.

**Conclusions:**

The sensitivity of only 67% of our standard workup emphasizes the difficulty to adequately diagnose low-grade infections after shoulder arthroplasty. The excellent specificity of 95% ensures, however, that non-infected prostheses are not incorrectly explanted. This study highlights that synovial biopsy has a high diagnostic value and should be done prior to complex revision surgeries to raise sensitivity in diagnosing a PJI.

**Supplementary Information:**

The online version contains supplementary material available at 10.1007/s00402-021-03932-x.

## Introduction

A common reason for painful total shoulder arthroplasties (TSA) and revision surgery is a periprosthetic joint infection (PJI) [27]. The mean incidence in primary TSA has been reported to be about 1% [22, 30]. Risk factors for PJI are especially posttraumatic osteoarthritis, previous surgery, rheumatoid arthritis, diabetes mellitus, and male sex [26, 27, 30, 31, 35].

Low-grade PJI are generally difficult to detect [32]. In contrast to acute PJI (usually < 4 weeks after implantation), low-grade PJI rarely show inflammatory symptoms [33]. The typical causal spectrum of microorganisms is composed of slowly growing bacteria such as *Cutibacterium* acnes and coagulase-negative skin pathogens *Staphylococcus* sp. (e.g. *S. epidermidis*, *S. capitis*, *S. haemolyticus*) [24, 26, 27]. In addition, standard diagnostic measures such as joint aspiration are often inconclusive, and in many cases—in contrast to other joints like the knee or hip—not possible due to punctio sicca [14].

The diagnosis of a PJI is of great importance preoperatively, because it plays a crucial role in determining the further therapeutic strategy, for example, if revision can be performed as single-stage or two-staged surgery with total explantation [12]. It is thus of paramount importance to identify a PJI preoperatively instead of having unexpected positive cultures which occurs in up to 23.9% of revision surgeries of TSA [21]. Identification of the pathogen is also essential to administer the correct systemic and local antibiotic therapy at the time of revision surgery [12].

To detect a PJI several specific and unspecific methods are available [10]: unspecific methods are radiographic imaging, C-reactive protein (CRP) concentration and erythrocyte sedimentation rate in blood, white blood cell count in blood and in synovial fluid, α-Defensin levels and leukocyte esterase presence in synovial fluid [5, 8, 19, 29], and histopathological grading of the synovial membrane [16, 18]. A specific method is the microbiological cultivation of preoperatively obtained synovial fluid through joint aspiration or preoperative biopsy. Microbiological cultivation allows to identify the causative micoorganism and its sensitivity to antibiotic treatment [3, 7, 12, 15, 17].

To define a PJI, in 2011 the musculoskeletal infection society (MSIS) proposed a series of major and minor diagnostic criteria, which were adapted in 2014. Major criteria are two positive periprosthetic cultures of aspirated joint fluid and/or synovial tissue samples with phenotypically identical organisms as well as a fistula communicating with the joint. Minor criteria are elevated serum CRP level and elevated erythrocyte sedimentation rate, elevated synovial fluid white blood cell count or positive reaction by leukocyte esterase test strips, elevated polymorphonuclear neutrophil percentage in the joint fluid, a positive histological result of the periprosthetic tissue and a single positive culture of periprosthetic tissue or fluid. According to the diagnostic criteria of the MSIS, the existence of a low-grade PJI is proven if one major criterion or at least three of the five minor criteria are met [23].

Specifically for shoulder PJIs, the 2018 International Consensus Meeting (ICM) introduced a scoring system that includes three further categories (probable, possible, and unlikely PJI) in addition to the original criteria with the intention to be able to estimate the probability of a PJI [13].

Specific evidence is, however, lacking for most criteria for a PJI of the shoulder. Estimates are mostly drawn from the hip or knee where the physiopathology is in some aspects different. Previous studies on the knee and hip prostheses have shown a sensitivity of 82–100% and a specificity of 98–100% through biopsy, which is thus superior to joint aspiration or the determination of CRP value in blood [9, 11, 25]. This also seems to hold true regarding a PJI of the shoulder where a sensitivity of only 7% for white blood cell count and of 25% for CRP-levels in serum have been reported [32]. Even fluoroscopically guided glenohumeral aspiration yielded only a sensitivity of 16.7% [6]. In line with the results from knee and hip arthroplasty, one study analysing 19 patients with a PJI of the shoulder suggested a very high diagnostic value for preoperative biopsy [6].

In everyday clinical practice, it is not only the diagnostic value of an individual method that is important but also the value of a diagnostic workup preceding revision surgery. In our present study, we evaluated the value of the preoperative synovial biopsy in the context of the performance of the entire diagnostic workup. We hypothesized that the highest sensitivity and specificity would be found in the biopsy technique.

## Materials and methods

### Patient collective

We retrospectively analysed 56 out of 73 patients who had undergone revision surgery of their TSA. Inclusion criterion was a revision operation of a TSA, the indication of which was based on clinical, radiological, laboratory and histological findings. Exclusion criteria were the presence of an early infection (previous operation < 4 weeks), early postoperative complications (previous operation < 4 weeks) and the lack of a histological sample from the definitive revision operation. Revision surgeries took place between December 2009 and April 2019. All patients were treated in the Markgröningen Orthopedic Clinic.

All patients presented at consultation due to problems with their TSA and all subsequently underwent our diagnostic workup. This study was approved by the local ethics board of the university hospital of Tuebingen (registration number 675/2019BO2).

### Preoperative diagnostic workup

**Fig. 1:**
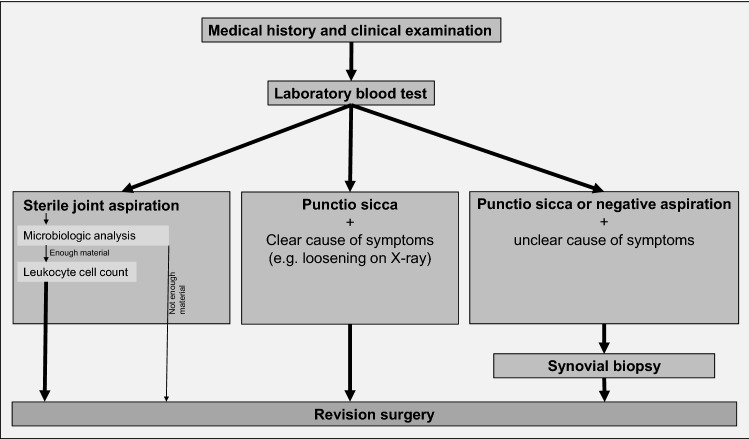
Flowchart of the preoperative diagnostic workup

To diagnose or to rule out a PJI, all patients were subjected to a diagnostic workup. Taking a medical history and clinical examination was followed by a laboratory blood test analysing CRP and white blood cell count. Furthermore, a sterile aspiration of the shoulder joint under fluoroscopic control was performed in all patients. If material could be obtained, a microbiological sample was taken. If, thereafter, still fluid was available the leukocyte cell count was measured. If the aspiration produced a punctio sicca or a negative result, but the symptoms (pain, limited range of motion) had no clear cause (e.g. humeral or glenoid loosening on X-ray, periprosthetic fracture, glenoid wear), a biopsy was performed (Fig. 1).

### Microbiological and histological methods

Microbiological and histological testing was performed as previously described [9, 11]. No patient had received antibiotics in the 4 weeks prior to aspiration or biopsy.

Joint aspiration and biopsy were always performed under sterile conditions and fluoroscopic guidance accessing the joint from ventral with the patient supine and the affected arm slightly externally rotated and abducted. The needle was inserted without local anesthesia 1 cm caudal and lateral to the tip of the coracoid process in a slightly medial direction. If fluid was harvested, it was immediately injected into vials containing BD BACTEC-PEDS-PLUSTM/F-Medium (Becton Dickinson, Heidelberg, Germany). A minimum of 1–3 ml was necessary for further analyses. The culture vials were treated with a growth enhancer (Fastidious Organism Supplement (FOS), Becton Dickinson) according to the manufacturer’s instructions and incubated using the BD BACTECTM 9050 automatic blood culture system (Becton Dickinson). Cultures were considered negative if no growth occured after 14 days according to previous studies [28].

Biopsies were always carried out in the operating room under general anesthesia. They were performed under fluoroscopic guidance using a biopsy forceps via an arthroscopic anterior portal. Five microbiological and five histological samples were obtained from the synovial lining of the humeral and glenoid zones. Arthroscopy was not performed to avoid a dilution effect on the samples.

When all samples had been obtained, a prophylactic single dose of cefazolin was administered perioperatively. The samples were placed in sterile tubes, transferred to the microbiologic laboratory within one hour and processed immediately. Each sample was processed separately. Microbiological analyses of the gained tissue samples were performed essentially as previously described [2, 34]. Cultures were checked daily and considered negative if no growth occured within 14 days. Microorganisms were identified by standard procedures including biochemical characterisation with the API system (BioMerieux, Nuertingen, Germany).

### Interpretation of the diagnostic workup

Each test was evaluated individually and it was decided whether it speaks for (positive) or against (negative) a PJI.

Laboratory blood tests were classified as positive with elevated CRP-levels (> 5 mg/l) or elevated white blood cell count in blood (> 9000/μl). In joint aspirate, a positive result was considered to be present in case of detection of bacteria as described above or an elevated white blood cell count (> 1100/μl). A biopsy sample was microbiologically positive with two positive periprosthetic cultures of synovial tissue with phenotypically identical organisms. Histological samples were regarded as positive if at least five polymorphonuclear leukocytes per high power field (400× magnification) were identified in one of ten such fields. The results of the individual tests were then combined and evaluated in accordance with the MSIS 2014-criteria described above. The MSIS criteria were used instead of ICM 2018-criteria because the collection of data starts 2009 and data registration was based on its suitability to the MSIS criteria.

Definitive classification as a PJI or not was based on diagnostic microbiological samples from the revision surgery. Both the entire workup and each individual diagnostic tool were then compared with the microbiological evidence in the revision operation. A sample was regarded microbiologically positive with two positive periprosthetic cultures of synovial tissue with phenotypically identical organisms. Only histological evidence in the case of negative microbiology was not counted as an infection, as this is a minor criterion. The tissue samples harvested during revision underwent the same bacteriological and histological analyses as the biopsy samples. Contamination of the samples from the revision surgery by preoperative aspiration or biopsy cannot be ruled out, but due to the antiseptic precautionary measures described above, it is very unlikely.

### Statistical analysis

Statistical analysis was conducted using IBM SPSS Version 20 (IBM, Armonk, NY, USA) and Microsoft Excel (Microsoft, Redmond, WA, USA). Distributions of variables within the groups were assessed by histograms and a non-parametric approach was chosen. Continuous variables are presented as medians and ranges, and categorical variables as frequencies. Comparison between groups was performed by Mann–Whitney *U* test or Chi-squared test as appropriate.

Values for sensitivity, specificity, positive predictive and negative predictive value and the accuracy are reported and their 95% confidence interval (CI) is given. All reported *p* values are two-sided, with a significance level of 0.05, and have not been adjusted for multiple testing.

## Results

Of the 56 patients, only two presented with discrete local clinical signs of infection (swelling, redness). No patient showed any sign of an accompanying fistula. Median patient age was 69 years (range 42–82) with 39 women and 17 men. Median BMI was 30 kg/m^2^ (range 20–58). The composition of the revised prostheses consisted of 20 inverse, 12 anatomical and 24 hemiprostheses (see Supplementary file 1). The most common primary diagnoses that had initially led to the implantation of an endoprosthesis were osteoarthritis and fracture (Table 1).

**Table 1:** Primary diagnoses that had initially led to the implantation of an endoprosthesis

Primary diagnosis	Number of patients (*n* = 56)
Osteoarthritis	30
Fracture	18
Avascular necrosis of the humeral head	4
Defect arthropathy	2
Rheumatoid arthritis	1
Resection of the humeral head	1

The most common symptoms at the first consultation were pain and limited mobility. The median time from primary operation (implantation of TSA) and the median time from the preceding operation (primary implantation or revision surgery) to this initial presentation was 27 months (range 2–150) and 24 months (range 0–150), respectively.

The diagnosis of a low-grade PJI determined by the results obtained from revision surgery was formulated in 15 of 56 cases (prevalence 27%). In 13 out of these 15 cases, only one microorganism was identified (Table 2).

**Table 2:** Identified microorganisms or identified combination of microorganisms

Identified microorganisms or identified combination of microorganism	Number of cases
*Cutibacterium* acnes	7
*Staphylococcus epidermidis*	5
*Staphylococcus capitis*	1
*Staphylococcus hominis* sap hominis/*Propionibacterium granulosum*	1
*Pseudomonas aeruginosa*/*Enterococcus faecalis*/*Staphylococcus epidermidis*	1

Common reasons for revision surgery in cases without PJI were mechanical complications (especially prolonged painful movement), glenoid dislocation or aseptic loosening (Table 3).

**Table 3:** Non-infectious reasons for the diagnostic workup prior to revision surgery

Reason	No PJI	PJI
Mechanical complication	11	–
Glenoid dislocation	8	–
Aseptic loosening	5	–
Chronic dislocation	5	3
Glenoid wear	4	–
Rotator cuff rupture	4	–
Periprosthetic fracture	3	1
Acute dislocation	1	–

*PJI* periprosthetic joint infection

The laboratory blood tests showed very limited diagnostic value. The number of elevated CRP values as well as the mean leukocyte count did not differ significantly between groups (Table 4).

**Table 4:** Increased CRP values and mean leukocyte count in blood

Variable	No PJI	PJI	*p* value
Increased CRP values (> 5 mg/l)	18/41 (43.9%)	9/15 (60.0%)	0.286*
Mean leukocyte count in blood	7458/μl (SD 2219)	7960/μl (SD 1910)	0.198°

*PJI* periprosthetic joint infection, *CRP* C-reactive protein

^*^Chi-squared test

°Mann–Whitney *U* test

Twenty-seven of 56 aspirations (48%) remained punctio sicca. As a result, diagnostics from joint aspirate such as the detection by microbiological culture or the white blood cell count can not be assessed on a regular basis. Due to the limited amount of fluid from joint aspiration after sending out the microbiological probes only in 16 cases still the white blood cell count could be determined. In the group with a PJI one out of four cases (25%) had actually an elevated white blood cell count in the synovial fluid. In the group without PJI, 22% had elevated values.

Since a microbiological analysis was carried out whenever an aspirate could be obtained, a result was received in 29 cases. No single microbiologically positive result was found.

Twenty-two of the 56 cases were biopted. The biopsy was positive in 30% and 80% in the group with PJI for histological and microbiological analyses, respectively. The histological and microbiological biopsy combined was positive in nine out of ten (90%) cases in the group with PJI. Of the twelve cases without PJI, ten cases were true negative and two cases were false positive.

The values of the different single diagnostic methods and the combination of these are shown in Table 5.

**Table 5:** Diagnostic values of the different diagnostic methods

Statistical parameter	Leukocytes in blood	CRP value in blood	Microbiology in aspirate	White blood cell count in aspirate	Biopsy (histological and micro-biological combined)	Diagnostic algorithm	Diagnostic algorithm without biopsy	Diagnostic algorithm in cases with biopsy
True positives (number)	5/56	9/56	0/29	1/16	9/22	10/56	0/56	10/22
True negatives (number)	32/56	23/56	21/29	9/16	10/22	39/56	41/56	10/22
False positives (number)	9/56	18/56	0/29	2/16	2/22	2/56	0/56	2/22
False negatives (number)	10/56	6/56	8/29	4/16	1/22	5/56	15/56	0/22
Sensitivity (95% CI)	33.3% (11.8–61.6)	60% (32.3–83.7)	0% (0–36.9)	20% (0.5–71.6)	90% (55.5–99.8)	66.7% (38.4–88.2)	60% (0–21.8)	100% (69.2–100)
Specifity (95% CI)	78.1% (62.4–89.4)	56.1% (39.75 –71.5)	100% (83.9–100)	81.8% (48.2–97.7)	83.3% (51.6–97.9)	95.1% (83.5–99.4)	100% (91.4–100)	83.33% (51.6–97.9)
Positive predictive value (95% CI)	35.7% (18.2–58.2)	33.4% (22.6–46.2)	–	28.7% (4.5–77.7)	66.4% (35.4–87.7)	83.3% (55.3–95.3)	–	66.7% (38.3–88.6)
Negative predictive value (95% CI)	76.2% (68.3–82.6)	79.3% 66.1–88.3)	73.2% (73.2)	73.6% (62.4–82.4)	95.8% (77.7–99.3)	88.6% (79.2–94.1)	72.4% (72.4–72.4)	100%
Accuracy (95% CI	66.1% (52.2–78.2)	57.1% (43.2–70.3)	73.2% (53.6–87.8)	65.3% (38–86.7)	85.12% (63.6–96.5)	87.5% (75.9–94.8)	72.4% (58.8–83.5)	87.8% (66.9–97.2)

*CRP* C-reactive protein, *CI* confidence interval

### Diagnostic value of the overall workup and the individual diagnostic tests

Our diagnostic workup showed a sensitivity of 67% (95% CI 38.4–88.2%). Specificity was 95% (95% CI 84–99%). Positive predictive value and negative predictive value were 84% (95% CI 55–95%) and 89% (95% CI 79–94%), respectively (Table 5). Noteworthy, without taking the synovial biopsy in account, no single PJI could be diagnosed according to the MSIS-criteria resulting in a sensitivity of 0% and a specificity of 100%.

When only looking at those cases that had additionally undergone diagnostic biopsy and when applying the MSIS-criteria, there was a sharp increase in sensitivity to 100% (95% CI 69–100%). Specificity had a slight decrease to 83% (95% CI 52–98%). Positive predictive value and negative predictive value were 67% (95% CI 38–89%) and 100%, respectively.

There were no complications in connection with the diagnostic methods performed. In particular, the aspiration and biopsy did reportedly not result in bleeding, post-operative infections or wound healing problems in any patient.

## Discussion

Of our analysed 56 cases of revised TSAs, 15 patients were revised for PJI.

Our diagnostic workup shows a high specificity (95%) with only a moderate sensitivity (67%). This sensitivity must be discussed against the background of the difficult-to-detect PJI of TSA [32]. Common causes of PJI are pathogens such as *Cutibacterium* acnes, a slow-growing microorganism that is difficult to detect [24, 26, 27]. A great advantage of the high specificity is the low probability of accidentally treating an uninfected prosthesis as infected. In our opinion, a lower sensitivity in favor of a higher specificity can be accepted for smaller revision surgeries of TSAs, especially since low-grade PJI in the shoulder do usually not present as a fulminant life-threatening clinical picture. However, for complex revision surgeries with high morbidity there is a need for a higher sensitivity, which can be achieved by means of synovial biopsy as presented in our study. Our hypothesis was that the preoperative biopsy has the greatest diagnostic value in the diagnostic workup. This hypothesis can be accepted with a sensitivity of 90% and a specificity of 83% for the biopsy alone. When performing a detailed analysis of these diagnostic biopsies, it can be noted that the microbiological analysis reached a sensitivity of 80% while the histological examination only yielded a sensitivity of 30%. Both modalities taken together reach the sensitivity of 90%. It needs to be pointed out here that the histopathologic interpretation of the tissue was simply based on counting polymorphonuclear leukocytes. This can be attributed to the retrospective character of the study with the earliest patients included from 2009. In the past few years, more differentiated techniques have been proposed that apparently allow for a more precise histopathological statement with respect to a PJI [16, 18]. We have also already adapted this system in our hospital. Although our first impression of the obtained results is very promising, we can not provide sufficient data yet to comment on it here. Another advantage of biopsy is the possible identification of the causative microorganism, so that resistance-appropriate antibiotic therapy can be administered immediately after or during the revision procedure [12].

Of note, when we only consider those cases who underwent the complete diagnostic algorithm, that means including minor criteria and biopsy, the sensitivity rises drastically to 100%. As already suggested and proven by similarly high sensitivities [4, 6], the biopsy appears to give a very reliable statement with respect to the presence of a low-grade infection of TSA. While sensitivity increases through the biopsy, specificity lowers (here 83% due to two false-positive results). It is, therefore, essential to keep the risks of potential contamination of the biopsy samples to an absolute minimum.

Common diagnostic methods such as laboratory blood test and aspiration had a rather frustrating diagnostic value in our study. The determination of the CRP value resulted in a sensitivity of 60% with a specificity of 53%, which is lower than in other joints but in line with recent studies concerning a PJI of TSA [1, 9, 11]. One possible reason is the slow-growing microorganisms, which typically do not cause a fulminant infection with elevated parameters of infection [32].

Moreover, especially in low-grade infections of the shoulder very little or no aspirate can be obtained. Punctio sicca occurred in 48% in our collective, which is comparable with other studies [14]. In primary cases of punctio sicca, irrigation with saline solution seems not to be promising [14]. Further synovial fluid analyses are, therefore, often not possible. If the material could be obtained, a microbiological sample was sent in first. If the material was enough for further tests, the number of white blood cells was measured. With a sensitivity of only 20%, the determination of the white blood cell count in the aspirate was surprisingly low and of little diagnostic value. One key reason may be again the lacking availability of the material. In this context, it makes also sense that its specificity of 82% appears to be quite reliable. Especially in low-grade infections the number of white blood cells in the synovial fluid has gained more attention recently with suggested limits indicative for infection being drastically lowered to values such as 3000/µl [13, 20, 23], depending also on the joint [20]. Microbiological culture from joint aspirate does not seem to provide sufficient diagnostic information: With 0% sensitivity not a single infection could be diagnosed by this method. Other studies investigating the sensitivity of microbiological examination of joint aspirate present a highly heterogeneous picture ranging in sensitivity from 7 to 81%. These sensitivities need to be seen in the context of the specific joint examined and the present microbiological spectrum [6, 14, 15]. Overall, however, the diagnostic value of aspiration must be classified as low.

A key limitation is the retrospective character of the study with its well-known weaknesses. A prospective study with the implementation of all diagnostic means would be desirable but has so far been difficult to carry out with the low frequency of the condition of a PJI of TSA and only just beginning acceptance of the biopsy. This also accounts for the small collective compared to studies on arthroplasty of the lower extremities, which is also reflected in the high ranges of sensitivity and specificity. To address this problem the scientific community to date still depends on review articles [4] or meta-analyses.

One key finding of our study is that the biopsy with microbiological and histological analyses combined has the highest diagnostic value in our diagnostic workup. Another key finding is that common dignostic methods have not enough diagnostic value to be used alone. We, therefore, strongly advocate to include synovial biopsy in the standard preoperative diagnostic workup.

## Conclusion

PJI of TSA are very difficult to detect. Since they are usually caused by microorganisms that lead to a low-grade infection, clinical symptoms are often unspecific and common diagnostic methods have only limited diagnostic value. Based on the data from our study, the diagnostic method with the highest diagnostic value is a synovial biopsy with microbiological and histological examination. In view of the low morbidity of synovial biopsy and its high diagnostic gain with direct consequences on further treatment of the patient, we recommend such biopsy in at least these cases where the common diagnostic workup is inconclusive.

## Supplementary Information

Below is the link to the electronic supplementary material.

Supplementary file1 (DOCX 13 KB)
