# Supplementary material for: The significance of synovial biopsy in the diagnostic workup of the low-grade periprosthetic joint infection of shoulder arthroplasty
Source: Arch Orthop Trauma Surg. 2021 May 15;142(11):3157–64. doi: 10.1007/s00402-021-03932-x (PMC9522757; doi:10.1007/s00402-021-03932-x)
Supplement: Supplementary file 1 — Supplementary file1 (DOCX 13 KB) [file 402_2021_3932_MOESM1_ESM.docx]

**Table 4:** Epidemiological information of the study collective (PJI: periprosthetic joint infection, BMI: body mass index)

|  | **Total** | **No PJI** | **PJI** |
| --- | --- | --- | --- |
| **Age**  **(years)** | 69  (range 42 – 82) | 71  (range 45 - 82) | 66  (range 42 - 76) |
| **Sex ratio**  **(w:m)** | 39 : 17 | 32 : 9 | 7 : 8 |
| **Weight**  **(kg)** | 81  (range 53 - 140) | 80  (range 53 – 140) | 86  (range 60 – 128) |
| **Height**  **(m)** | 1.64  (range 1.48 – 1.89) | 1.64  (range 1.48 – 1.89) | 1.67  (range 1.4 – 1.83) |
| **BMI** | 30  (range 20 – 58) | 29  (range 20 - 58) | 33  (range 23 - 40) |
| **Revised Prothesis** | 20 inverse  12 anatomical  24 hemiprostheses | 5 inverse  4 anatomical  6 hemiprotheses | 15 inverse  8 anatomical  18 hemiprotheses |
